# Validity of self-reported night shift work among women with and without breast cancer

**DOI:** 10.5271/sjweh.4142

**Published:** 2024-04-01

**Authors:** Jesper Medom Vestergaard, Jesper Nikolai Dietrich Haug, Annett Dalbøge, Jens Peter Ellekilde Bonde, Anne Helene Garde, Johnni Hansen, Åse Marie Hansen, Ann Dyreborg Larsen, Mikko Härmä, Sadie Costello, Henrik Albert Kolstad

**Affiliations:** 1 Department of Occupational Medicine, Danish Ramazzini Centre, Aarhus University Hospital, Aarhus, Denmark.; 2 Department of Occupational Medicine, Danish Ramazzini Centre, University Research Clinic, Goedstrup Hospital, Herning, Denmark.; 3 Institute of Clinical Medicine, Aarhus University, Denmark.; 4 Department of Occupational and Environmental Medicine, Bispebjerg and Frederiksberg Hospital, Denmark.; 5 The National Research Centre for the Working Environment, Denmark.; 6 Department of Public Health, University of Copenhagen, Denmark.; 7 Danish Cancer Institute, Danish Cancer Society, Denmark.; 8 Finnish Institute for Occupational Health, Finland.; 9 Environmental Health Science, School of Public Health, University of California, Berkeley, USA.

**Keywords:** case, control, gold standard, misclassification, patient, validation study

## Abstract

**Objectives:**

This study aimed to estimate the validity of self-reported information on ever-night shift work among women with and without breast cancer and illustrate the consequences for breast cancer risk estimates.

**Methods:**

During 2015–2016, 225 women diagnosed with breast cancer and 1800 matched controls without breast cancer employed within the Danish hospital regions during 2007–2016 participated in a questionnaire-based survey. Their reported night shift work status was linked with objective payroll register day-by-day working hour data from the Danish Working Hour Database and the Danish Cancer Registry. For the breast cancer patients and their matched controls, we estimated sensitivity and specificity for ever working night shifts using the payroll data as the gold standard. We also used quantitative bias analysis to estimate the impact on relative risk estimates for a hypothetical population.

**Results:**

For breast cancer patients, we observed a sensitivity of ever-night shifts of 86.2% and a specificity of never-night shifts of 82.6%. For controls, the sensitivity was 80.6% and the specificity 83.7%. Odds ratio for breast cancer in a hypothetical population decreased from 1.12 [95% confidence interval (CI) 1.03–1.21] to 1.05 (95% CI 0.95–1.16) when corrected by the sensitivity and specificity estimates.

**Conclusion:**

This study shows that female breast cancer patients had slightly better recall of previous night shift work than controls. Additionally, both breast cancer patients and controls recalled previous never-night shift work with low specificity. The net effect of this misclassification is a small over-estimation of the relative breast cancer risk due to night shift work.

In 2007, the International Agency for Research on Cancer (IARC) concluded that shift work involving circadian disruption and, in 2020, that night shift work are probably carcinogenic to humans (Group 2A) (1, 2). The latter conclusion was based on sufficient evidence in experimental animals for the carcinogenicity of alteration in the light–dark schedule, strong evidence in experimental systems that alteration in the light–dark schedule exhibits key characteristics of carcinogenesis, and limited evidence in humans for the carcinogenicity of night shift work (2). The strongest epidemiological evidence was, according to the IARC evaluation, seen in case–control studies of breast cancer (2). The largest case–control study, a pooled analysis of five case–control studies by Cordina-Duverger et al (3), reported an overall odds ratio (OR) of 1.12 [95% confidence interval (CI) 1.00–1.25] for breast cancer among women who ever worked night shifts. The Nurses’ Health Study, a large prospective follow-up study, showed a two-fold – but no overall – increased risk of breast cancer among participants who were young at the time of enrollment and had worked rotating night shifts for ≥20 years or more (4). Most studies included in the IARC evaluation of breast cancer relied on self-reported data on night shift work through face-to-face interviews (5–7), in-person questionnaires (8–10), self-administered questionnaires (4, 11) or a combination thereof (3).

Differential exposure misclassification is a potential challenge in case–control studies relying on recall of previous exposures as cases may tend to identify possible reasons for their disease contrary to healthy controls (12). Non-differential misclassification of exposure may bias results of case–control as well as follow-up studies (13, 14). Therefore, validation studies and quantitative bias analysis are important to understand and evaluate the magnitude of such biases (15). We compared, for the first time, the validity of self-reported night shift work among women with and without breast cancer and assessed the impact on breast cancer risk estimates.

## Methods

### Population

The Danish Working Hour Database (DWHD) provided information on every female employee (N=206 894) from every Danish public hospital in all five regions with information on day-by-day working hours from payrolls from 1 January 2007 (four regions) and 1 January 2008 (one region), or the first day of employment if later, until 31 December 2015, or last date of employment if earlier (16). In 2015–2016, 48 909 currently employed female workers in three of the five regions were invited to participate in an e-mail-based survey on working hours and related topics (17). A total of 29 497 employees (60.7% among breast cancer patients and 60.3% among potential controls) responded and 27 438 (93%) provided complete information on night shift work and alcohol consumption for further analyses. Most workers also reported height and weight used to calculate body mass index and smoking status.

### Breast cancer patients and controls

Data on breast cancer was obtained from the Danish Cancer Registry, which keeps records on all cancers diagnosed in Denmark since 1943. A total of 225 women participants were diagnosed with first time breast cancer (ICD-10: C50) after their first year of employment (as recorded in DWHD, ie, 2008/2009) and before the date of participation in the survey, and were included in the analyses. The first time restriction was because at least one previous calendar year with employment information was needed for night shift work status classification (as defined later). We denote the calendar year when breast cancer was diagnosed as the “index year”.

For each breast cancer patient, we randomly selected eight matched controls without breast cancer (the maximum number available given the matching criteria) with replacement among the potential controls. A participant was a potential control for a specific breast cancer patient if she was not diagnosed with breast cancer, had the same age and reported the same alcohol consumption (<3 versus ≥3 units/week) as the breast cancer patient at the time of the survey. Furthermore, she should have the same night shift work status (ever- versus never-night shift work as defined below) as recorded in DWHD prior to the index year to have a balanced distribution of night shift work among patients and controls. In total 1800 controls (1717 unique individuals) were selected.

### Night shift work

**Figure 1:**
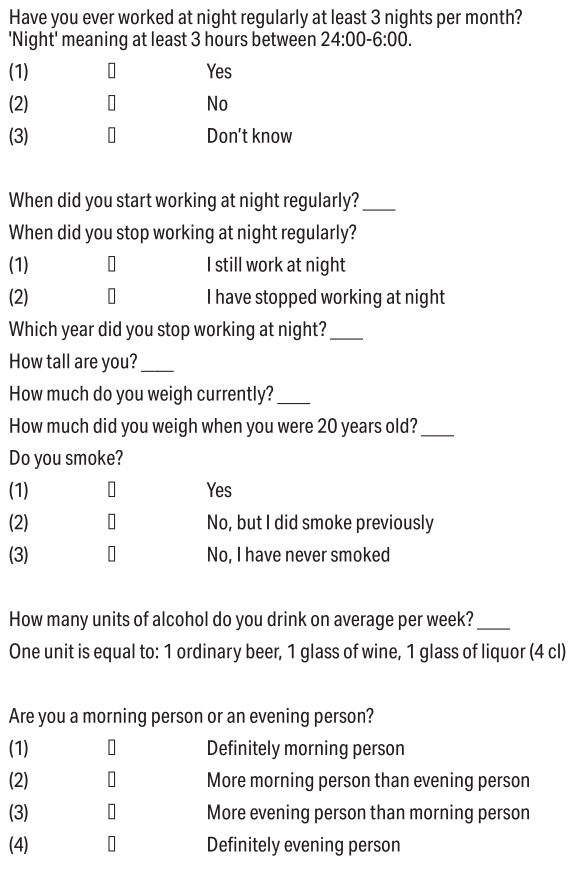
2015-2016 questionnaire on previous night shift work and lifestyle

A night shift was defined as ≥3 hours of work between 24:00 and 06:00 hours. Night shift work was classified as ever-night shift work that was defined as ever≥1 month with ≥3 night shifts from first recorded year of employment until and including the year before the index year, else as never-night shift work. Our definition was comparable with that used in the Nurses’ Health Study except that it did not require day- or evening shifts in addition to the night shifts within a month (4). We decided on this definition because the Nurses’ Health Study has provided several highly influential results on health effects of night shift work (4, 18, 19). According to the DWHD data, 94.8% of cases classified with ever-night shift work in the current study population had rotating night shift work as defined in the Nurses’ Health Study. For controls, the figure was 90.0%. The definition of night shift work applied equally to survey and payroll register data. The brief survey questionnaire is shown in figure 1.

DWHD provided information on occupation. Survey data, breast cancer diagnosis, and DWHD data were linked at individual level by the unique personal identification number that all residents of Denmark are applied.

According to Danish law, studies based entirely on registry and questionnaire data do not require approval from an ethics review board. All questionnaire participants gave informed consent. The analysis was registered at the repository of the Central Denmark Region (j. no: 1–16-02–653-18), and the Danish Health Data Authority approved data access (707394, FSEID-00004107 and FSEID-00004926).

### Statistical methods

We estimated sensitivity (probability of true ever-night shift work) and specificity (probability of true never-night shift work) of self-reported compared with register-based night shift work, which we considered the gold standard. We computed 95% CI using 100 bootstrap datasets, each based on a sample with replacement of the 225 breast cancer patients and their matched controls. We calculated the difference in sensitivity and specificity between breast cancer patients and controls.

We furthermore conducted a quantitative bias analysis for a hypothetical population comparing the observed risk estimate for breast cancer following ever-night shift work to the risk estimates obtained after correcting the night shift work misclassification by the sensitivity and specificity estimates (20). The hypothetical population included 6000 breast cancer cases and 6000 controls, had an exposure prevalence among the controls as in our study population and a risk estimate for breast cancer following night shift work as in the Cordina-Duverger et al (3) pooled study which included 6093 breast cancer cases and 6933 breast cancer free controls. Analyses were conducted using Stata version 17 (StataCorp, College Station, TX, USA) and the Excel spreadsheet of Lash, Fox and Fink (20).

## Results

Participants with ever-night shift work were younger than those with never-night shift work, consumed less alcohol, were more often never smokers and primarily employed as physicians or nurses (table 1). Only 58 (26%) breast cancer patients were identified as having worked night shifts in accordance with the definition in the DWHD (table 2). The same proportion (26%) was seen in controls because of the matching.

**Table 1:** Characteristics of breast cancer patients and matched controls among Healthcare workers, Denmark 2007–2016.

Characteristics		Breast cancer patients				Controls		
		Never-night shift work ^a^		Ever-night shift work ^a^		Never-night shift work ^a^		Ever-night shift work ^a^
		N=167		N=58		N=1336		N=464
		N (%)		N (%)		N (%)		N (%)
Age (years)								
	<50	40 (24.0)		21 (36.2)		320 (24.0)		168 (36.2)
	50–54	44 (26.3)		17 (29.3)		352 (26.3)		136 (29.3)
	55–59	40 (24.0)		14 (24.1)		320 (24.0)		112 (24.1)
	≥60	43 (25.7)		6 (10.3)		344 (25.7)		48 (10.3)
Alcohol units per week on average								
	<3	86 (51.5)		36 (62.1)		688 (51.5)		288 (62.1)
	≥3	81 (48.5)		22 (37.9)		648 (48.5)		176 (37.9)
Body mass index (kg/m^2^)								
	<25 (normal weight)	96 (57.5)		31 (54.4)		793 (59.9)		268 (58.1)
	25–<30 (overweight)	45 (26.9)		21 (36.8)		365 (27.6)		128 (27.8)
	≥30 (obese)	26 (15.6)		5 (8.8)		165 (12.5)		65 (14.1)
Smoking								
	Current	13 (7.8)		4 (6.9)		126 (9.5)		45 (9.8)
	Previous	85 (51.2)		22 (37.9)		535 (40.2)		170 (37.0)
	Never	68 (41.0)		32 (55.2)		669 (50.3)		245 (53.3)
Occupation								
	Physicians	6 (3.6)		9 (15.5)		62 (4.7)		41 (8.8)
	Nurses and midwifes	54 (32.3)		38 (65.5)		486 (36.6)		287 (61.9)
	Auxiliary nurses, janitors and orderlies	28 (16.8)		8 (13.8)		175 (13.2)		94 (20.3)
	Other	79 (47.3)		3 (5.2)		606 (45.6)		42 (9.1)
Index year ^b^								
	2008–2012	78 (46.7)		28 (48.3)		624 (46.7)		224 (48.3)
	2013–2016	89 (53.3)		30 (51.7)		712 (53.3)		240 (51.7)

^a^ Never-night shift work and ever-night shift work defined by payroll data. ^b^ Calendar year the breast cancer patient was diagnosed with breast cancer, split in two groups by the median.

**Table 2:** Sensitivity and specificity of self-reported versus payroll register night shift work for women breast cancer patients and their matched controls, Denmark, 2007–2016. [CI=confidence intervals, in this case based on 100 bootstraps.]

		Breast cancer patients						Controls				
		Payroll register						Payroll register				
		Ever-night shift work			Never-night shift work			Ever-night shift work			Never-night shift work	
		N	% (95% CI)		N	% (95% CI)		N	% (95% CI)		N	% (95% CI)
Self-reported												
	Ever-night shift work	50	86.2 (77.3–95.1)		29	17.4 (11.2–23.6)		374	80.6 (76.9–84.3)		218	16.3 (14.3–18.3)
	Never-night shift work	8	13.8 (4.9–22.7)		138	82.6 (76.4–88.8)		90	19.4 (15.7–23.1)		1118	83.7 (81.7–85.7)

Of 58 breast cancer patients, 50 reported ever-night shift work in agreement with our gold standard register data, corresponding with a sensitivity of 86.2% (95% CI 77.3%–95.1%) (table 2). The corresponding sensitivity for controls was 80.6% (95% CI 76.9%–84.3%). The specificity was 82.6% (95% CI 76.4%–88.8%) for breast cancer patients and 83.7% (95% CI 81.7%–85.7%) for controls. The differences in sensitivity and specificity were 5.6% (95% CI -4.8%–16.0%) and -1.1% (95% CI -7.4%–5.2%) when comparing breast cancer patients with controls.

The quantitative bias analysis of the hypothetical population with an exposure prevalence of 26% among the controls and an odds ratio of 1.12 showed a corrected OR of 1.05 (95% CI 0.95–1.16) (table 3).

**Table 3:** Observed and corrected odds ratios of night shift work and breast cancer from quantitative bias analysis of a hypothetical population. [CI=confidence intervals.]

	Observed					Corrected ^a^			
	Cases	Controls	Odds ratio	95% CI		Cases	Controls	Odds ratio	95% CI
Ever-night shift work	1695	1560				946	905		
Never-night shift work	4305	4440	1.12	1.03–1.21		5054	5095	1.05	0.95–1.16
Total	6000	6000				6000	6000		

^a^ Corrected numbers calculated from sensitivity and specificity estimates of night shift work obtained among breast cancer patients and matched controls employed within 3 of 5 Danish hospital regions, 2007–2015, Denmark, using Excel spreadsheet of Lash, Fox and Fink (20).

## Discussion

This study of primarily hospital employees observed a slightly higher sensitivity of self-reported ever-night shift work among breast cancer patients (86.2%) than among matched controls without breast cancer (80.6%) when compared with objective payroll information on night shift work. This study also observed low specificity among breast cancer patients and controls, showing that both groups had difficulties classifying themselves correctly as never-night shift workers in the survey.

Our suggestive finding of better recall of previous night shift work among breast cancer patients compared to their matched controls is a concern because this pattern of differential misclassification tends to inflate risk ratio estimates (12, 14). On the other hand, low specificity of exposure classification tends to deflate risk ratio estimates. The quantitative bias analysis of the hypothetical population showed that the net effect of such differential and non-differential misclassification produced a corrected risk ratio estimate that was slightly lower (OR=1.05) than the naive estimate (OR=1.12). This finding underpins the importance of considering both types of exposure misclassification when interpreting results of epidemiological studies and the strength of quantitative bias analysis when a gold standard is available (20). It has to be emphasized that our results relate to the current (or a similar) study population and may not be generalizable to other study populations with a different prevalence of night shift work.

### Comparison with other studies

Härmä et al (21) observed a 96% sensitivity and a 92% specificity of self-reported “shift work with night shifts” among hospital employees when compared with individual-level payroll records. The higher sensitivity and specificity compared with ours is likely due to a wider formulation of the questions used in the survey (and consequently the definitions of the payroll data). They defined night shift work by a question stating “*What is your usual work schedule?*”, with “*Shift work with night shifts*” being one of five response options. We used the following question: “*Have you ever worked at night regularly at least 3 nights per month? Nights meaning at least 3 hours between 24:00–06:00*”. The questions used in the Härmä study may have allowed for a more accurate classification of night shift work compared to our questions, which were much narrower. Härmä did not consider validity related to breast cancer status. Lizama et al (22) observed that breast cancer patients more often than controls believed that shift work increase the risk of breast cancer, but they did no formal evaluation of misclassification. We are not aware of other studies validating self-reported night shift work.

Quantitative bias analysis can offer valuable insight into the impact of exposure misclassification; however, there are few examples in the occupational literature. Notably, Deltour (23) showed lower risk estimates of acoustic neuroma after correcting self-reported occupational noise exposure using quantitative bias analysis. Biased recall of other occupational exposures have been assessed without conducting a formal bias analysis of the net-effect of differential and non-differential recall (23–26), leaving the field unsure of the impact of the misclassification on the reported risk ratio estimate.

### Limitations and strengths

Our study population had to survive for up to eight years (median three) from the index year and remain employed within the five hospital regions to participate. Even if 91% of breast cancer patients in the total DWHD population returned to work within half a year, this may have affected our validity estimates compared with estimates based on self-reports obtained with a short lag (the case for most case–control studies). It is unclear if this lag will affect breast cancer patients and controls differently. Further, the participation proportion in our survey was only about 60% and might be skewed compared to the entire population.

The availability of payroll data for only recent night work (2007–2015) and only covering employment within the five hospital regions are also limitations. The skewed distribution of physicians and nurses between breast cancer patients and controls could be a problem if they have better (or worse) memory of night shift work than the other occupations. Unfortunately, the limited number of breast cancer patients did not allow matching on occupation.

The small number of breast cancer patients with ever-night shift work resulted in uncertain estimates of sensitivity that can only be solved with a larger study population, a broader night shift work definition, or a higher prevalence of night shift work.

The main strengths of this study were the concurrent availability of self-reported and detailed register-based payroll information on working hours, the latter collected prior to breast cancer diagnosis and a nationwide and virtually complete cancer registry, making it possible to compare the sensitivity and specificity by breast cancer status.

In conclusion, this study of Danish female hospital employees shows that breast cancer patients slightly better recall previous ever-night shift work compared to controls while both breast cancer patients and controls recall previous never-night shift work with low specificity. The net effect of this misclassification is expected to be a small over-estimation of the relative risk of breast cancer following night shift work for a study conducted in a similar population and using a similar, singular, night shift work survey question.
